# The iADRS as an integrated measure of cognition and function: Psychometric evidence from recent clinical trials in early symptomatic Alzheimer's disease

**DOI:** 10.1002/alz.70656

**Published:** 2025-09-14

**Authors:** Alexandra S. Atkins, Sarah Marquis, Kristin Creel, Juliette Meunier, Stefan Cano, Laure Delbecque, Mingyang Shan, Alette M. Wessels

**Affiliations:** ^1^ Eli Lilly and Company Lilly Corporate Center Indianapolis Indiana USA; ^2^ Modus Outcomes, (a THREAD Company) Cary North Carolina USA

**Keywords:** Alzheimer's disease, CDR, crosswalk, iADRS, MMSE, RMT

## Abstract

**INTRODUCTION:**

Psychometric evaluation of the integrated Alzheimer's Disease Rating Scale (iADRS) was performed using data from three randomized clinical trials (RCTs).

**METHODS:**

Traditional measurement properties of the iADRS were assessed using classical test theory (CTT). Rasch Measurement Theory (RMT) analyses evaluated response properties of cognitive and functional items across the disease severity continuum. Crosswalks between iADRS, Clinical Dementia Rating – Sum of Boxes (CDR‐SB), and Mini‐Mental State Examination (MMSE) were developed to provide estimates of score equivalence.

**RESULTS:**

CTT results confirm good psychometric properties of the iADRS, including reliability (Cronbach's alpha 0.79–0.84), concurrent validity with CDR‐SB (range: −0.64–0.70), and sensitivity to change. RMT findings demonstrate improved reliability of the integrated scale (person‐separation index [PSI] = 0.92) compared to components and highlight concurrent and overlapping progression of cognitive and functional decline.

**DISCUSSION:**

The findings confirm the psychometric strength of the iADRS as a fit‐for‐purpose integrated endpoint for RCTs in early symptomatic Alzheimer's disease (AD). Crosswalk tables facilitate interpretation of clinical trial findings in clinical practice.

**Highlights:**

- Findings confirm integrated Alzheimer's Disease Rating Scale (iADRS) total score reliability, validity, and sensitivity.
- Using the Rasch Measurement Theory (RMT), iADRS item set shows improved reliability relative to its component subscales.
- iADRS item thresholds are well distributed along the disease severity continuum.
- iADRS shows sensitivity to concurrent progression of cognitive and functional impairment.
- Crosswalk tables provide direct translation between iADRS, Clinical Dementia Rating – Sum of Boxes (CDR‐SB), and Mini‐Mental State Examination (MMSE).

## 1 INTRODUCTION

Alzheimer's disease (AD) is a progressive neurodegenerative disorder characterized by insidious declines in cognition and daily function that progress heterogeneously over many years, ultimately leading to complete dependence and loss of life.^1^, ^2^, ^3^ Over the past decade, increased scientific consensus regarding underlying disease pathology and identification of reliable biomarkers sensitive to disease‐related pathology (e.g., amyloid beta, p‐tau) have contributed to an industry‐wide shift toward design of clinical trials targeting early symptomatic AD, inclusive of mild cognitive impairment (MCI) due to AD and mild AD dementia,^4^ paving the way for regulatory approval of the first disease modifying therapies in AD.^5^, ^6^

The integrated Alzheimer's Disease Rating Scale (iADRS) was developed in response to the need for clinical efficacy endpoints with improved sensitivity to clinical declines observed in early symptomatic AD. Development of the iADRS aligned with recommendations from the US Food and Drug Administration (FDA) on the development and use of integrated outcomes to better measure clinical decline in this patient population.^7^

Development and initial validation of the iADRS has been previously described in detail.^8^ Briefly, the iADRS incorporates 31 items from two widely accepted measures: the AD Assessment Scale‐Cognitive subscale (ADAS‐Cog_13_), assessing cognition, and the AD Cooperative Study‐Instrumental Activities of Daily Living (ADCS‐iADL), assessing daily functioning.^8^ The iADRS combines direct input from multiple sources (patient, clinician, and study partner) to provide a single measure of global disease severity across the continuum of early symptomatic AD.

In previous work, the iADRS demonstrated effective monitoring of disease progression and reliable detection of treatment effects in early symptomatic AD, supporting its use as a clinical efficacy endpoint in multiple phase 2 and 3 trials in AD.^9^, ^10^, ^11^, ^12^ Most recently, the iADRS was used to demonstrate clinical efficacy of donanemab,^13^ making it the second of two primary endpoints used in successful FDA submissions for disease modifying treatments (DMTs) for early symptomatic AD, the first being the CDR‐SB.

Although the Clinical Dementia Rating – Sum of Boxes (CDR‐SB) and iADRS both provide integrated assessment of cognition and function, there are marked differences between the two. While the CDR‐SB reflects the summation of six individual clinician ratings of cognition and function, the iADRS includes 31 items incorporating performance‐based cognitive testing (ADAS‐Cog test items), informant ratings of daily functioning (iADL), and clinical ratings (ADAS‐Cog clinical items) into a single endpoint. Given these differences, increased understanding of the measurement properties and performance of both measures is warranted, as is continued evaluation of novel approaches that may ultimately provide tools with increased sensitivity and precision supporting future clinical trials.

As such, the aim of the present work is to provide comprehensive psychometric evaluation of the iADRS instrument in early symptomatic AD using two distinct methodologies: classical test theory (CTT) and Rasch Measurement Theory (RMT). CTT analyses evaluate and describe standard measurement properties of the iADRS total score, including internal consistency reliability, construct validity, and ability to detect change. RMT methods^14^, ^15^, ^16^ aim to measure latent constructs through item responses, corresponding to the 1‐parameter model in item response theory (IRT). RMT analyses assess both the functioning of discrete items within a scale and their collective performance to measure the core concept of interest.

In this study, RMT was used to assess the measurement properties of the iADRS and to gain insight into the integration of cognitive and functional items within the scale. RMT was also used to better understand disease progression in AD through evaluation of the item response hierarchy, and to provide a way to compare the results from the iADRS with other comparable instruments. RMT analyses provided assessments of total score reliability, item‐to‐sample targeting, ordering of item difficulty (least to most difficult), and patient ability (most to least impaired) along a single linear scale representing the latent construct of global disease severity.

Finally, crosswalk tables were created that enable conversion of raw scores for the iADRS total, the Mini‐Mental State Examination (MMSE), and the CDR‐SB based on pooled RMT analyses across studies. By providing estimated score equivalence between commonly used measures that vary in administration methodology (e.g., interview‐based clinical ratings; performance‐based cognitive testing) and scoring systems, crosswalks offer a standardized method to equate and compare scores. This is particularly useful for interpreting findings from clinical studies using different endpoints (e.g., CDR‐SB; iADRS) and for applications in clinical practice.

## 2 METHODS

### 2.1 Study design and participants

Data from three double‐blind, placebo‐controlled studies were analyzed: the phase 2 TRAILBLAZER‐ALZ (NCT03367403) and phase 3 TRAILBLAZER‐ALZ 2 (NCT04437511) studies evaluated the safety and efficacy of donanemab for treatment of participants with early symptomatic AD,^10^, ^11^ and the phase 3 EXPEDITION‐3 trial (NCT01900665) evaluated the safety and efficacy of solanezumab in participants with mild AD.^9^ Data included all participants randomized to either placebo or active treatment with a baseline and at least one post‐baseline iADRS assessment; data were blinded with respect to treatment group assignment.

All trials were conducted in accordance with ethical principles of the Declaration of Helsinki and Good Clinical Practice guidelines. The research protocols were approved by each center's institutional review board or ethics committee. All participants provided written informed consent.

Clinical trial design and inclusion criteria have been described previously.^9^, ^10^, ^11^ Briefly, TRAILBLAZER‐ALZ and TRAILBLAZER‐ALZ 2 trials included participants aged 60–85 years with early symptomatic AD with MMSE scores of 20 to 28 and confirmed amyloid and tau pathology. Participants were randomized 1:1 to receive donanemab or placebo intravenously every 4 weeks for up to 72 weeks. The primary endpoint in the TRAILBLAZER‐ALZ trials was the change in the iADRS total score from baseline to 76 weeks in donanemab versus placebo‐treated groups.

The EXPEDITION‐3 study enrolled participants aged 55–90 years with mild AD, confirmed amyloid pathology and MMSE scores of 20 to 26. Participants were randomized 1:1 to receive intravenous solanezumab or placebo every 4 weeks for 76 weeks. The primary endpoint was the change in the ADAS‐Cog_14_ total score from baseline to 80 weeks in solanezumab versus placebo‐treated groups; the change in the iADRS total score from baseline to 80 weeks was a secondary endpoint.

**RESEARCH IN CONTEXT**

1. **Systematic review**: Literature review included prior reports on development, psychometric evaluation, and use of the integrated Alzheimer's Disease Rating Scale (iADRS). Analyses performed provide robust psychometric characterization of the iADRS as an integrated assessment of cognition and function.
2. **Interpretation**: Findings provide confirmation of the iADRS as a fit‐for‐purpose endpoint for evaluation of treatment effects in early symptomatic AD. The classical test theory (CTT) results confirm reliability, validity, and sensitivity of the iADRS total, while the Rasch Measurement Theory (RMT) findings show sensitivity to concurrent declines in cognition and function over the spectrum of disease severity. Crosswalks (conversion tables) between iADRS, Clinical Dementia Rating – Sum of Boxes (CDR‐SB), and Mini‐Mental State Examination (MMSE) facilitate clinical interpretation across measures.
3. **Future directions**: Present findings provide robust characterization of the iADRS in participants with early symptomatic Alzheimer's disease (AD) followed over 18 months. To enrich understanding of scale performance in later stages of AD, future work should incorporate longer observation periods and/or include a wider range of baseline disease severity.

### 2.2 Clinical outcome assessments

The following clinical outcome assessments (COAs) were included in CTT and RMT psychometric analyses:

#### 2.2.1 iADRS, ADAS‐Cog, and ADCS‐iADL

The iADRS integrates 31 items from two scales, the ADAS‐Cog_13_ (13 items assessing cognitive function) and the ADCS‐iADL (18 items assessing instrumental activities of daily living), to provide a single summary total score of global disease severity. The iADRS total score is calculated as a linear combination of the two component scales, reversing the score for the ADAS‐Cog_13_ and anchoring it as zero, as follows: ADCS ‐iADL + (85 – ADAS‐Cog_13_). The iADRS score ranges from 0 to 144 with lower scores indicating greater impairment. The first of the two component scales contributing to the iADRS is the ADAS‐Cog_13_, a rater‐administered instrument with both clinician‐reported and performance‐based items that assess areas of cognitive functioning most commonly impacted by AD.^17^ The ADAS‐Cog_13_ score ranges from 0 to 85, with higher scores indicating greater impairment. The second component of the iADRS is the ADCS‐iADL, an 18‐item subscale of the ADCS‐Activities of Daily Living (ADCS‐ADL)^18^ administered by a clinician as a structured interview with an informed study partner to assess the patient's ability to perform daily activities. The score ranges from 0 to 59, with lower scores indicating greater impairment.

Measurement properties of the iADRS were evaluated throughout initial scale development and testing of the instrument using historical data from several AD clinical trial populations.^8^, ^19^, ^20^ Previous research has established the content validity of the iADRS,^21^, ^22^ demonstrating convergent validity through moderate to strong correlations with the CDR‐SB, the MMSE, and the Functional Activities Questionnaire (FAQ)^8^, ^19^, ^20^ and identified thresholds for clinical meaningful within‐person change (MWPC) in early symptomatic AD.^23^ Analyses of qualitative data from the *What Matters Most (WMM)* initiative^21^, ^24^, ^25^ highlighted the iADRS as the clinical outcome assessment tool that provides the most comprehensive coverage of concepts identified as most meaningful for patients with early symptomatic AD.^21^

#### 2.2.2 CDR

The CDR assesses cognition and function, using semi‐structured clinical interviews completed separately with the patient and an informed caregiver/ study partner to gather information regarding the patient's current status in six domains including memory, orientation, judgment and problem solving, home and hobbies, community affairs and personal care.^26^ Following both interviews, the clinician assigns the patient a rating of 0 (no impairment), 0.5 (very mild impairment), 1 (mild impairment), 2 (moderate impairment), or 3 (severe impairment). The CDR‐SB score sums ratings across all six domains and ranges from 0 to 18, with higher scores indicating greater impairment. The CDR Global score provides clinical staging of dementia along the following scale: 0 (normal), 0.5 (very mild dementia), 1 (mild dementia), 2 (moderate dementia), and 3 (severe dementia).

Alternative staging using CDR‐SB has also been developed to allow for more fine‐grained examination of change over time.^27^ Staging categories of the CDR‐SB score are: 0 = normal, 0.5–4.0 = questionable cognitive impairment, 3.0–4.0 very mild dementia, 4.5–9.0 = mild dementia, 9.5–15.5 = moderate dementia, and 16.0–18.0 = severe dementia.^27^ As a continuous measure, the CDR‐SB comprises the primary endpoint in several prior ongoing randomized clinical trials (RCT)s. Despite ceiling effects on several individual CDR domains in early symptomatic AD, the total CDR‐SB score demonstrates ceiling effects within the acceptable range, as well as good sensitivity to decline, test–retest reliability, internal consistency reliability, and construct validity.^28^

Given widespread use and acceptance of the CDR‐SB and CDR Global scores for clinical assessment and staging of AD, both measures were used in CTT analyses to evaluate construct validity of iADRS. In addition, CDR‐SB was incorporated into crosswalk analyses providing estimated equivalence between scores on the CDR‐SB, MMSE, and iADRS.

#### 2.2.3 MMSE

The MMSE is a brief, rater‐administered assessment of global cognitive status.^29^ MMSE total scores range from 0 to 30, with higher scores indicating better cognitive performance. MMSE scores are utilized for broad staging of cognitive impairment as follows: 27–30 (questionably significant impairment), 20–26 (mild impairment), 10–19 (moderate impairment), and ≤9 (severe impairment).^30^, ^31^ A consensus of previous research shows that reliability and construct validity of the MMSE are satisfactory, but not all items are equally sensitive to cognitive impairment and scores are impacted by demographic characteristics.^32^

Given widespread use in research and clinical practice, the MMSE was used in CTT analyses and incorporated into crosswalk analyses providing direct translation between scores on the CDR‐SB, MMSE, and iADRS.

### 2.3 CTT analyses

The CTT analyses were performed separately for each study and included all participants randomized to either placebo or active treatment with a baseline and at least one post‐baseline iADRS assessment. Completeness of the iADRS total score was assessed at baseline and each post‐baseline visit.

Internal consistency reliability for the iADRS was assessed by computing Cronbach's alpha coefficient^33^ at baseline. Thresholds for interpretation of the coefficient were defined conservatively, based on recommendations of Nunnally et al.: <0.70 = unsatisfactory reliability; 0.70–0.79 = modest reliability; 0.80–0.89 = adequate reliability, and 0.90–1.00 = good reliability.^34^

Construct validity was evaluated using Spearman correlation to confirm the association between the iADRS, the CDR‐SB and the MMSE at baseline according to predefined hypotheses. The iADRS total score was expected to show moderate negative correlation (Spearman's |*r*|≥0.3) with both the CDR‐SB and MMSE scores. Additionally, the baseline mean iADRS scores were compared across categories of the CDR‐SB and MMSE (both described above) using analysis of variance (ANOVA).

Ability of the iADRS to detect change over time was assessed by examining the change in iADRS total score from baseline to end of study (week 76 for TRAILBLAZER‐ALZ trials and week 80 for the EXPEDITION‐3 trial). Established MWPC values for the CDR‐SB and MMSE^35^, ^36^, ^37^ were used to define anchors in CTT analyses of iADRS change over time. Importantly, while MWPC thresholds comprise FDA's recommended method for evaluating clinical meaningfulness of individual patient‐level change on a given outcome,^38^ they are not appropriate thresholds for interpretation of treatment effects defined based on group comparisons between treatment arms.^37^, ^38^, ^39^ Furthermore, as empirically defined metrics, MWPC values may not always align with alternative views on clinical meaningful change, such as those endorsed by clinical consensus.

Participants were classified as improved, stable, or worsened based on MWPC values for the CDR‐SB (≤−2, between −2 and 2, and ≥2, respectively) and MMSE (≥2, between −2 and 2, and ≤‐2, respectively).^35^, ^36^ These thresholds were based on MWPC ranges provided by Landsall et al.^36^, ^37^, ^40^ and Andrews et al.;^35^ a 2‐point change threshold for change on each measure was selected based on AD staging of the population of interest. Kazis’ effect sizes (ES)^41^ for change in iADRS scores between baseline and end of study were computed within each subgroup (improved, stable, and worsened). Spearman correlations were calculated between change on the iADRS and change on the CDR‐SB, CDR global score, and MMSE from baseline to end of study period.

### 2.4 RMT analyses

The main objective of the RMT analyses were to evaluate the extent to which the iADRS endpoint reflects integrated assessment cognition and function across the range of disease severity observed. Other objectives included investigating the relationship between decline on cognitive and functional items and comparing the iADRS with other measures of cognition and daily function. RMT analyses were conducted using the iADRS as well as the component measures, the ADAS‐Cog_13_ and the ADCS‐iADL, to examine the integration of these two concepts of interest.

RMT methods focus on the relationship between a person's level of ability (in this case, cognitive and functional ability) and the difficulty of items within a scale. RMT analyses examine the extent to which the observed data (individual item responses) fit item response predictions from the Rasch model, which defines how a set of items should perform to generate reliable and valid measurements.^14^ RMT methods use a mathematical model (the Rasch model) to evaluate the legitimacy of summing items to generate an interpretable score.^16^ More specifically, RMT analyses examine the extent to which the observed data (actual responses to scale items) fit predictions of the responses expected from the Rasch model, which defines how a set of items should perform to generate reliable and valid measurements.^14^ The difference between expected and observed scores indicates the degree to which rigorous measurement is achieved, with smaller differences indicating better model fit. Through the Rasch model, a metric is established and calibrated by estimating item parameters known as item thresholds. Item response thresholds, comparable to increments on a ruler, represent points of measurement that correspond to shifts in expected responses to the items; mean item thresholds, referred to as item locations, represent the average response threshold for each item on the RMT‐defined logit scale. Participants are mapped to a location on this same RMT metric based on scale performance across all iADRS items. RMT analyses were performed with the unrestricted polytomous model for ordered responses.^15^ Participants with missing visits were included in the scheduled assessments for which they were present. RMT was performed in RUMM2030 (RUMMLab Pty Ltd).^42^

#### 2.4.1 Targeting

A key psychometric property examined in the ADAS‐Cog_13_, ADCS‐iADL, and iADRS was targeting, which refers to the match between the distribution of the concept of interest (cognitive and functional ability) in the patient sample and the range of the concept of interest measured by the individual items in the instrument. A better match between patient sample and item levels results in more stable item calibration, and more precise measurement of person abilities. Analyses of targeting included comparing the spread, in logits, of participant ability and item difficulty, comparing the mean participant ability to the mean item difficulty, which is set to 0, and finally, evaluating the information curve, which indicates where, in logits, the scale has the most precision (more specifically, where participant scores have the smallest standard errors).

#### 2.4.2 Assessment of the iADRS item set as a measurement continuum

Another psychometric property examined for the ADAS‐Cog_13_, ADCS‐iADL, and iADRS was an assessment of each item set as a measurement continuum via investigation of (i) functioning of response options (examination of the ordering of response thresholds), (ii) item hierarchy, (iii) item fit, and (iv) local dependence. Item fit was evaluated using fit residuals and chi‐squared statistics to examine the extent to which the items conform as a set to measure the intended concept. Comparable values in both fit residuals and chi squared statistics imply a conforming set of items; items with outlying estimates merit further investigation to explain the misfit. Local dependence, which tests if items are more closely related to each other rather than to the concept of interest, was examined using residual correlations between item pairs, with a desired threshold <0.3.

#### 2.4.3 Reliability

Reliability was examined using a person‐separation index (PSI), a statistic comparable to Cronbach's alpha, evaluated as >0.7 good, >0.8 very good, and >0.9 excellent.

#### 2.4.4 Test characteristic curves

Test characteristic curves were drawn to examine the relationship between cognition and daily function across the measurement continuum.

#### 2.4.5 Analyses of the iADRS components

Two anchored RMT analyses were performed to characterize the progression of cognitive and functional impairment over a single measurement continuum defined by the iADRS. First, items and response options from the ADCS‐iADL (assessing daily function) were mapped to a single metric defined by the items of the ADAS‐Cog13 (ADCS‐iADL anchored to ADAS‐Cog13). In the second, items and response options from the ADAS‐Cog13 (assessing cognition) were mapped to a single metric defined by the items of the ADCS‐iADL (ADAS‐Cog13 anchored to ADCS‐iADL).

#### 2.4.6 Crosswalk

Finally, a crosswalk, or conversion table, was derived between the iADRS, the CDR‐SB, and the MMSE to allow for direct comparison between total scores. The crosswalk was developed by first performing a grouped RMT analyses with all three measures (iADRS, CDR‐SB, and MMSE) and then on each individual measure using anchored item parameters from the common metric established by the grouped analysis. Then, equating was performed by matching a raw score from one test to the closest score from another test on the latent scale. A crosswalk was established by interpolating values when the variation in score ranges mapped to multiple scores on another measure. Validation was performed by randomly splitting data 80:20 into training and validation data sets, respectively. The RMT model was fit and the crosswalk estimated using the training data set; intraclass correlation coefficients (ICCs) and their 95% confidence intervals (CIs) with absolute (type A,1) agreement^43^ were computed for the validation data set. A threshold of ≥0.70 was considered adequate for group level comparisons.^34^, ^44^

## 3 RESULTS

### 3.1 CTT analyses

#### 3.1.1 Participants

Participant demographic and disease characteristics are summarized by study in Table 1. Across trials, mean age was approximately 73 years and just over half of participants were female. Participants in the two TRAILBLAZER‐ALZ studies generally had more years of formal education than in the EXPEDITION‐3 study. Baseline clinical scores were similar across the three studies. The mean CDR‐SB score at baseline was 3.46, 3.92, and 3.90 for TRAILBLAZER‐ALZ, TRAILBLAZER‐ALZ 2, and EXPEDITION‐3, respectively, all of which fall into the CDR‐SB staging category of very mild dementia. The mean MSME score at baseline was 23.67, 22.33, and 22.73 for TRAILBLAZER‐ALZ, TRAILBLAZER‐ALZ 2, and EXPEDITION‐3, respectively, all of which fall into the MMSE staging category of mild impairment. Total iADRS scores at baseline were 106.18, 103.98, and 103.91 for TRAILBLAZER‐ALZ, TRAILBLAZER‐ALZ 2, and EXPEDITION‐3, respectively.

**TABLE 1:** Baseline demographics and disease characteristics of participants in TRAILBLAZER‐ALZ, TRAILBLAZER‐ALZ 2, and EXPEDITION‐3.

Parameter	TRAILBLAZER‐ALZ (*N*=245)	TRAILBLAZER‐ALZ 2 (*N*=1687)	EXPEDITION‐3 (*N*=2124)
Age in years, mean (SD)	75.09 (5.46)	72.95 (6.17)	72.96 (7.89)
Female, *n* (%)	125 (51.0%)	964 (57.1%)	1226 (57.7%)
Years of education, *n* (%)			
No formal education	0	6 (0.4%)	0
<9 years	4 (1.6%)	78 (4.6%)	276 (13.0%)
10–12 years	49 (20.0%)	395 (23.4%)	610 (28.7%)
≥13 years	192 (78.4%)	1207 (71.5%)	1235 (58.1%)
Missing	0	1 (0.1%)	3 (0.1%)
Time in years since AD diagnosis, mean (SD)	1.75 (1.84)	1.46 (1.78)	1.55 (1.64)
Baseline CDR‐SB score, mean (SD)	3.46 (1.88)	3.92 (2.07)	3.90 (1.93)
Baseline MMSE total score, mean (SD)	23.67 (2.95)	22.33 (3.88)	22.73 (2.84)
Baseline iADRS total score, mean (SD)	106.18 (12.86)	103.98 (14.10)	103.91 (13.89)

Abbreviations: AD, Alzheimer's disease; CDR‐SB, Clinical Dementia Rating—Sum of Boxes; iADRS, integrated Alzheimer's Disease Rating Scale; MMSE, Mini‐Mental State Examination; SD, standard deviation.

#### 3.1.2 Completeness of the iADRS

For TRAILBLAZER‐ALZ, TRAILBLAZER‐ALZ 2, and EXPEDITION‐3, respectively, completeness of the iADRS total score at baseline was 100%, 97.3%, and 99.9%. The iADRS total score was available for at least 98% of participants at every visit in TRAILBLAZER‐ALZ and was available for over 90% of participants in TRAILBLAZER‐ALZ 2 and EXPEDITION‐3 at most visits, indicating good and consistent completion of the measure.

#### 3.1.3 Reliability

Within each trial, Cronbach's alpha of the iADRS total score was evaluated at baseline as a measure of internal consistency reliability. Cronbach's alpha values were 0.84, 0.84, and 0.79 for TRAILBLAZER‐ALZ, TRAILBLAZER‐ALZ 2, and EXPEDITION‐3, respectively. All exceeded the minimum acceptable threshold of 0.70,^34^ indicating the iADRS demonstrated internal consistency reliability across all three trials.

#### 3.1.4 Construct validity

Spearman correlation coefficients indicated moderate‐to‐strong correlations between the iADRS total score and CDR‐SB and MMSE at baseline, which aligned with a priori hypotheses. For TRAILBLAZER‐ALZ, TRAILBLAZER‐ALZ 2, and EXPEDITION‐3, respectively, correlation coefficients were −0.66, −0.70, and −0.64 for the CDR‐SB and 0.55, 0.65, and 0.53 for the MMSE. Across all three trials, higher iADRS total scores were observed at baseline among participants with lower disease severity according to categories of both the CDR‐SB and MMSE (*p* < 0.0001; Table S1).

#### 3.1.5 Ability to detect change

Effect sizes (ES) were calculated for the mean change in iADRS total score between baseline and end of the study period according to categories defined by the changes in CDR‐SB and MMSE; results are shown in Table 2. Across the three trials, large ES (|ES|>0.80) were observed for participants who had ≥2‐point worsening from baseline in the CDR‐SB and MMSE. Small ES (0.2 ≤|ES|<0.5) were seen in stable participants (< = 2‐point change from baseline in CDR‐SB or MMSE). ES for participants who improved (> = 2‐point improvement from baseline on CDR‐SB or MMSE) were small to negligible (|ES|<0.31).

**TABLE 2:** ES for the change in iADRS total score between baseline to end of the study period according to improvement or worsening groups as defined by the CDR‐SB and MMSE, in TRAILBLAZER‐ALZ, TRAILBLAZER‐ALZ 2, and EXPEDITION‐3.

	TRAILBLAZER‐ALZ (*N* = 245)			TRAILBLAZER‐ALZ 2 (*N*‐1687)			EXPEDITION‐3 (*N* = 2124)		
Parameter	*N*	ES	Mean change (SD)	*N*	ES	Mean change (SD)	*N*	ES	Mean change (SD)
**CDR‐SB change from baseline**									
**Improved** (≤ ‐2 points change)	3	−0.11	−0.33 (4.16)	33	0.21	2.70 (12.59)	27	−0.27	−3.07 (11.23)
**Stable** (‐2 points ≤ change ≤ +2 points)	103	−0.40	−3.95 (7.39)	666	−0.41	−4.79 (8.41)	974	−0.47	−5.77 (10.59)
**Worsened** (≥ +2 points change)	77	−1.40	−16.74 (12.31)	529	−1.30	−17.03 (14.48)	776	−1.64	−22.60 (18.00)
**MMSE change from baseline**									
**Improved** (≤ ‐2 points change)	17	−0.31	−3.18 (5.13)	154	−0.15	−1.79 (8.55)	189	−0.11	−1.30 (9.16)
**Stable** (‐2 points ≤ change ≤ +2 points)	50	−0.47	−4.74 (7.49)	388	−0.38	−4.78 (8.89)	438	−0.37	−4.37 (9.48)
**Worsened** (≥ +2 points change)	112	−1.01	−11.85 (12.83)	681	−1.13	−14.49 (13.95)	1111	−1.34	−18.17 (17.05)

*Note*: Change was evaluated between baseline and week 76 in the TRAILBLAZER trials and between baseline and week 80 in EXPEDITION‐3.

Abbreviations: CDR‐SB, Clinical Dementia Rating ‐ Sum of Boxes; ES, standardized effect size; iADRS, integrated Alzheimer's Disease Rating Scale; MMSE, Mini‐Mental State Examination; SD, standard deviation.

Spearman coefficients indicated moderate‐to‐strong correlations between the decline in iADRS total score and changes in CDR Global, CDR‐SB, and MMSE scores between baseline and the end of each trial's study period. For TRAILBLAZER‐ALZ, TRAILBLAZER‐ALZ 2, and EXPEDITION‐3, respectively, coefficients were −0.42, −0.48, and −0.52 for CDR Global Score; −0.63, −0.57, and −0.60 for CDR‐SB; and 0.44, 0.51, and 0.62 for MMSE.

### 3.2 RMT analyses

#### 3.2.1 Targeting

**FIGURE 1:**
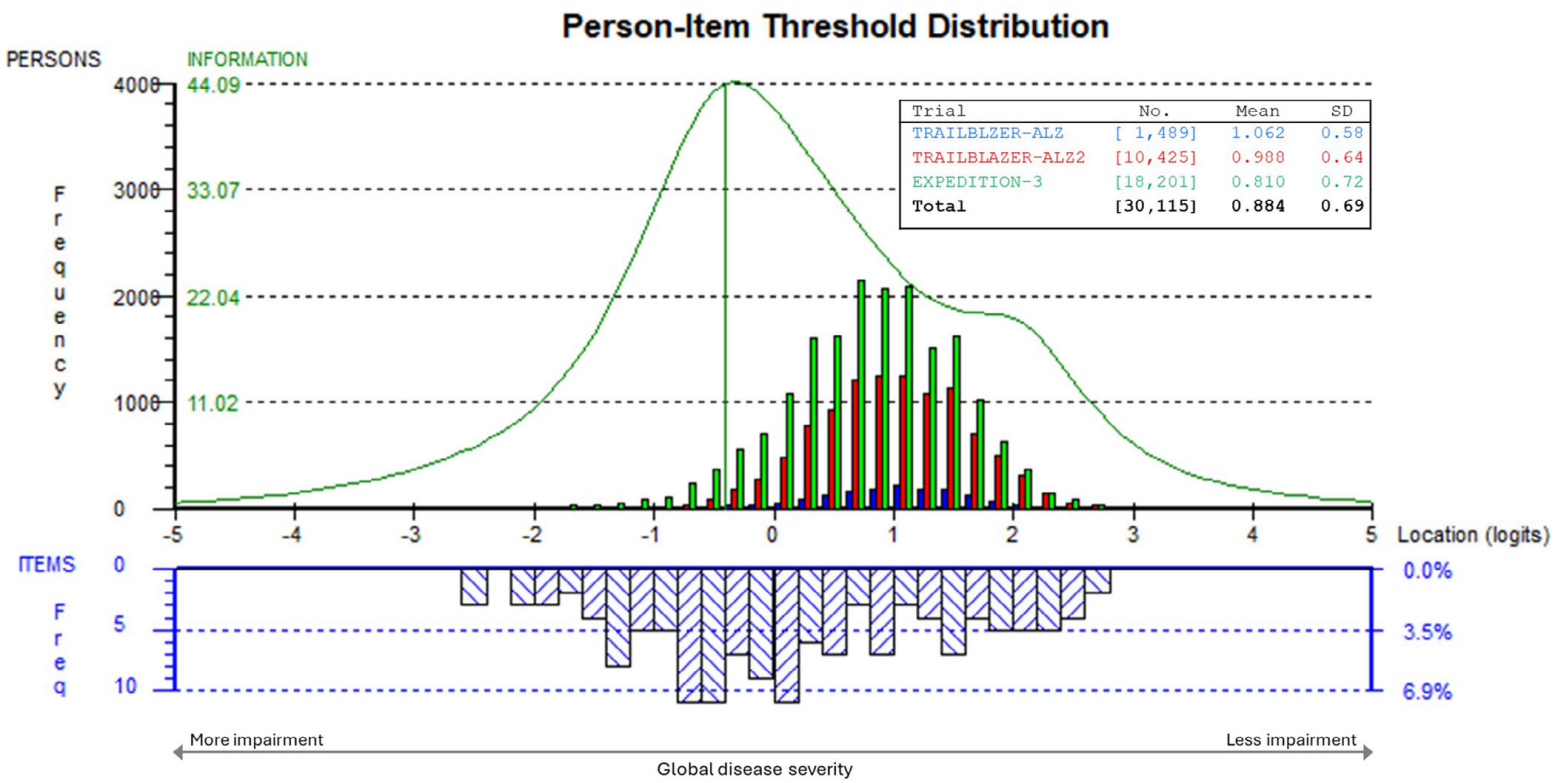
Targeting for the iADRS: distribution of observed patient ability levels versus item thresholds (blue)—using pooled and stacked data from TRAILBLAZER‐ALZ, TRAILBLAZER‐ALZ 2, and EXPEDITION‐3 studies. iADRS, integrated Alzheimer's Disease Rating Scale.

Targeting, which refers to the match between the distribution, in logits, of iADRS item difficulty and participant ability, as assessed by the iADRS total score, showed very good alignment in the pooled sample. As shown in Figure 1, item thresholds covered the full range of observed participant scores with no floor or ceiling effects. The participant ability for the pooled and stacked data (*N* = 30,115) was mostly distributed on the positive half of the latent scale represented by the iADRS, indicating an average cognitive and functional ability above the average item difficulty (mean person ability was 0.884, compared to the mean item difficulty, which is 0). The information curve (the green line in Figure 1) indicated that the part of the scale with the highest level of precision was aimed at patients somewhat more impaired than much of the sample. Individually, the three studies displayed similar distributions of impairment, with person ability means that were comparable for TRAILBLAZER‐ALZ (1.06) and TRAILBLAZER‐ALZ 2 (0.99) and slightly lower for EXPEDITION‐3 (0.81).

#### 3.2.2 Assessment of the iADRS item set as a measurement continuum

##### 3.2.2.1 Functioning of response options

For each item scored with successive integer responses (e.g., 1, 2, 3, and 4) representing differences in performance, the Rasch model hypothesizes a continuum of the underlying measured concept (e.g., AD disease severity) so that a change (increase or decrease) in score corresponds to a meaningful change in the measured concept.^45^ Ordered item response thresholds indicate that each change in item score (improvement or decline) reflects a change in patient disease severity in the expected direction. RMT analyses of the iADRS indicated that response categories (i.e., item scores) were correctly ordered for five cognitive items derived from the ADAS‐Cog_13_: *naming objects and figures* (Cog2), *commands* (Cog3), *orientation* (Cog6), *word‐finding difficulty in spontaneous speech* (Cog10), and *number cancelation* (Cog13). The remaining eight cognitive items showed potential limitations in functioning of directional scoring, implying that the sequential scoring of the response options was not fully consistent with the expected increase/decrease in the concept measured in the sample. This is not unexpected given that some items within the ADAS‐Cog_13_ have large score ranges, increasing the risk of deviation from fully ordered thresholds.

##### 3.2.2.2 Item hierarchy

The iADRS item hierarchy, determined by item locations (i.e, mean item response thresholds) on the logit scale, enabled an ordering of the items from most difficult (representing aspects of cognition and function that decline early in disease progression) to easiest (assessing aspects of cognition and function that decline latest). Table 3 provides the item hierarchy for iADRS items ordered from most difficult (DL20B) to least difficult (DL18C).

**TABLE 3:** iADRS item hierarchy (most‐to‐least difficult), established by placement of mean item response thresholds on the RMT logit scale, using pooled and stacked data from TRAILBLAZER‐ALZ, TRAILBLAZER‐ALZ 2, and EXPEDITION‐3.

Item	Statement	Mean location	Fit residuals	Chi‐square
DL20B	Talk about content of reading *after 1 h or more*	2.095	−6.02	30.46
Cog12	Delayed word recall	1.932	19.42	7.16
DL8C	Talk about TV content *within an hour*	1.611	−3.37	11.04
Cog13	Number cancellation	1.232	10.10	10.26
Cog7	Word recognition trial 1	1.184	78.00	118.86
Cog1	Word recall	1.106	−26.10	12.09
DL20A	Talk about content of reading *within an hour*	1.017	−14.23	10.15
DL21	Write things down	0.753	−2.67	15.00
DL7	Use a telephone	0.302	−0.98	6.36
DL16	Shopping	0.232	5.24	9.94
Cog6	Orientation	0.223	−0.27	3.05
DL13	Make a meal	0.104	4.03	0.52
DL8B	Talk about TV content *while watching*	0.088	−5.17	1.78
DL19B	Talk about outside events	0.075	−26.74	21.86
DL18A	Left away from home	0.022	−27.89	23.88
DL22	Perform pastime	−0.003	19.69	6.03
DL23	Use household appliance	−0.069	−4.38	5.83
DL11	Find belongings	−0.092	13.24	2.84
DL19C	Talk about at home events	−0.102	−23.90	20.73
DL17	Keep appointments	−0.144	−12.83	21.54
DL19A	Talk about events heard/read	−0.157	−20.20	15.30
Cog11	Remembering test instructions	−0.343	−5.18	1.85
DL6A	Usual dressing performance	−0.386	0.12	4.52
Cog5	Ideational praxis	−0.395	0.46	3.05
DL10	Clear dishes	−0.427	16.67	3.14
DL15	Get around outside home	−0.471	−9.29	10.87
Cog4	Constructional praxis	−0.493	12.10	6.85
DL9	Pay attention to conversation	−0.523	6.15	1.51
DL12	Obtain beverage	−0.552	5.38	3.16
DL8A	Select television program	−0.592	−13.63	10.87
DL14	Dispose of garbage	−0.595	2.57	1.84
Cog2	Naming objects and fingers	−0.629	−4.11	3.73
Cog3	Commands	−0.705	0.03	1.26
Cog10	Word finding difficulty in spontaneous speech	−0.735	4.91	2.23
DL18B	Left at home for 1 h or longer	−1.047	−14.31	13.93
Cog8	Spoken language ability	−1.048	−3.34	0.64
Cog9	Comprehension	−1.183	−10.66	3.17
DL18C	Left at home for less than 1 h	−1.288	−8.31	6.93

*Note*: Blue text = item from ADCS‐iADL, red text = item from ADAS‐Cog.

Abbreviations: iADRS, integrated Alzheimer's Disease Rating Scale; RMT, Rasch Measurement Theory.

iADRS items sensitive to impairment at the earliest stages of disease impairment included: talking about reading after 1 h or more (DL20B), delayed word recall (Cog12), talking about TV within an hour (DL8C), number cancellation (Cog 13), and word recognition (Cog7). iADRS items sensitive to impairment at the latest stages of observed disease severity included being left at home for less than one hour (DL18C), language comprehension (Cog9) and spoken language ability (Cog8), being left at home for longer than 1 h (DL18B), and word‐finding difficulty in spontaneous speech (Cog10). Cognitive and functional items falling in the middle of the hierarchy included constructional and ideational praxis (Cog4 and Cog5), remembering test instructions (Cog11), clearing dishes (DL10), paying attention to conversation (DL9), and keeping appointments (DL17).

**FIGURE 2:**
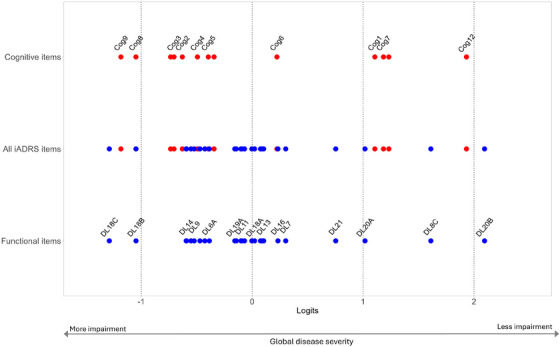
Mean iADRS item locations by cognitive items (ADAS‐Cog, in red) and functional items (ADCS‐iADL, in blue) using pooled and stacked data from TRAILBLAZER‐ALZ, TRAILBLAZER‐ALZ 2, and EXPEDITION‐3 (see Table 3 for iADRS item descriptions corresponding to each item label). ADAS‐Cog, AD Assessment Scale‐Cognitive subscale; ADCS‐iADL, AD Cooperative Study‐Instrumental Activities of Daily Living; iADRS, integrated Alzheimer's Disease Rating Scale.

Figure 2 plots item locations, calculated as mean item response thresholds, for iADRS items assessing daily function (in blue) and cognition (in red), demonstrating overlapping and interleaved coverage of cognitive and functional impairments across the measurement continuum. The distribution of daily function items and cognitive items in the iADRS (middle line) demonstrates that combining the two scales increases the coverage of items across the global disease severity continuum, resulting in a larger range and better coverage (See Table 3 for descriptions of each iADRS item label). Individual response thresholds for each iADRS item are provided in Table S2.

##### 3.2.2.3 Item fit

Evaluation of item fit using fit residuals and chi‐squared statistics in Table 3, above, identified to one item within the iADRS item set with clear outlying fit statistics: word recognition (Cog7). Results showed a similar  when the ADAS‐Cog13 and ADCS‐iADL were assessed individually, with all items demonstrating comparable chi‐squared statistics except for word recognition (Cog7), the only item to have a significant *p*‐value with chi‐squared statistics adjusted for sample size. iADRS fit residuals had a comparable range and distribution to the individual ADAS‐Cog13 and ADCS‐iADL analyses, pointing to the preservation of measurement properties in the integrated scale.

##### 3.2.2.4 Assessment of local independence assumption

Item residual correlations were above the recommended level of 0.3 for 12 of 703 pairs suggesting some local dependency among the items. The largest residual correlation was between being left at home less than one hour (DL18B) and being left at home more than 1 h (DL18C), *r* = 0.71. No local dependence was detected between cognitive (ADAS‐Cog13) and functional (ADCS‐iADL) items, providing supportive evidence for the integration of the ADAS‐Cog13 and ADCS‐iADL. Residual correlations for the iADRS are provided in supplemental materials, Table S3.

#### 3.2.3 Reliability

The PSI for the iADRS was 0.92, indicating excellent reliability and increased reliability as compared to the component scales, which was 0.88 for the ADAS‐Cog13 and 0.86 for the ADCS‐iADL, respectively.

#### 3.2.4 Test characteristic curves

**FIGURE 3:**
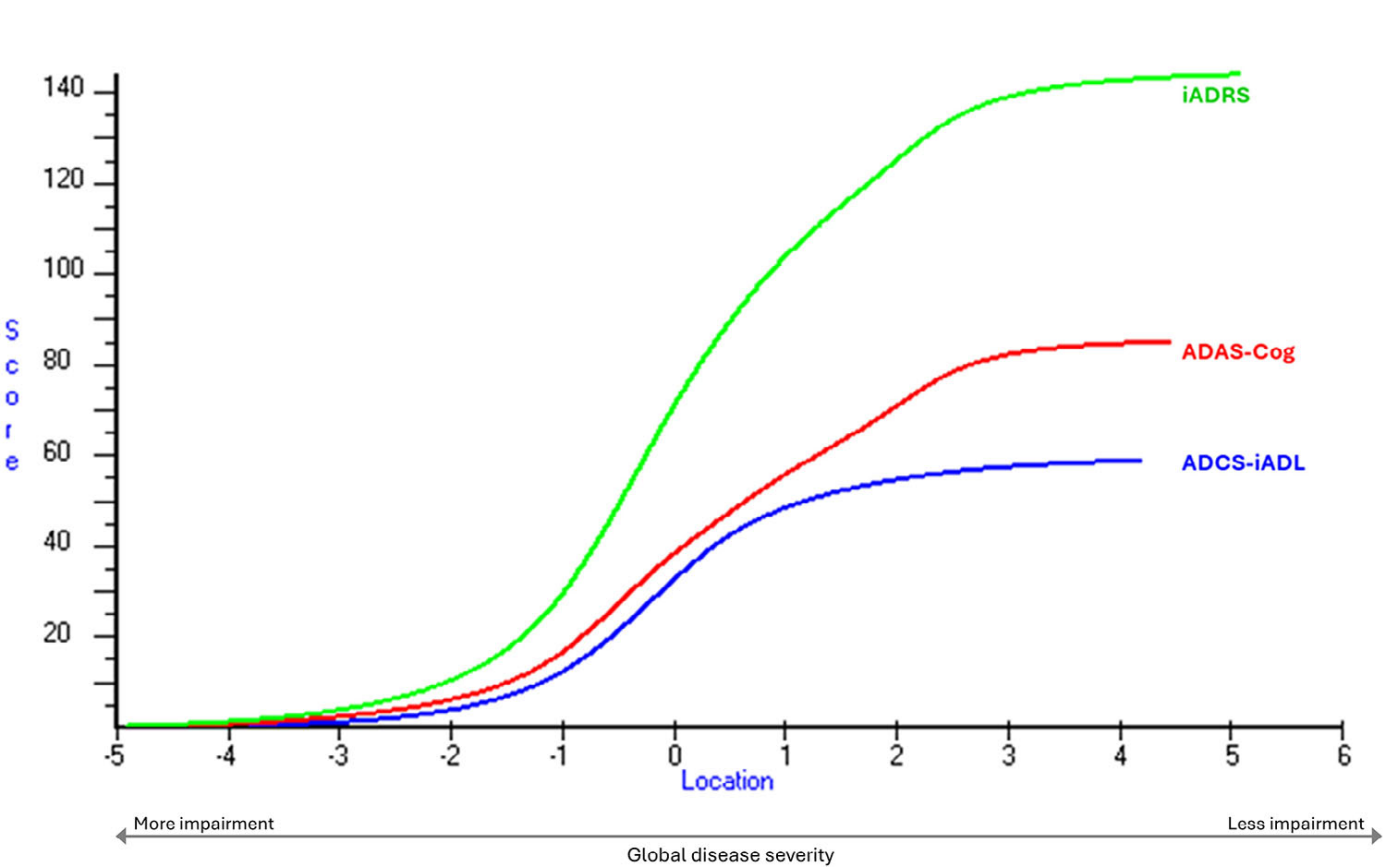
Expected progression of total scores for the iADRS, ADAS‐Cog, and ADCS‐iADL along the continuum of disease severity. ADAS‐Cog, AD Assessment Scale‐Cognitive subscale; ADCS‐iADL, AD Cooperative Study‐Instrumental Activities of Daily Living; iADRS, integrated Alzheimer's Disease Rating Scale.

Test characteristic curves for the iADRS and its component scales, the ADAS‐Cog13 and the ADCS‐iADL, provide expected total scores on each scale based on a patient's position on the disease severity continuum defined by item parameters calibrated in the RMT model. As shown in Figure 3, test characteristic curves for the ADAS‐Cog13 and ADCS‐iADL progress in parallel from −4 to 1 logits, indicating concurrent measurement of cognitive and functional performance in this range of disease severity. Although differences between final height of the ADAS‐Cog13 and ADCS‐iADL curves reflect difference in the score range of the two measures, separation of the two curves at approximately 1.5 logits suggests the earliest stage of disease severity was associated with a somewhat greater declines in cognitive performance compared to functional ability. This result is consistent with the slight ceiling effect observed on the ADCS‐iADL functional scale in the current study (Table 4) and with ceiling effects on functional domains of the CDR, described elsewhere.^28^ Importantly, the iADRS total score did not demonstrate ceiling or floor effects in the current population.

**TABLE 4:** Summary of RMT results using pooled and stacked data from TRAILBLAZER‐ALZ, TRAILBLAZER‐ALZ 2, and EXPEDITION‐3.

	ADAS‐Cog	ADCS‐iADL	iADRS
Parameter	13 items in RMT analyses	25 items in RMT analyses	38 items in RMT analyses
**Targeting**			
Ceiling/floor effect	None	Ceiling effect	None
Sample mean (SD)	0.97(0.74)	1.12(1.19)	0.88(0.69)
Chi‐squared	10097.6	11811.5	26148.7
DF	117	225	342
*p*‐value	<0.001	<0.001	<0.001
**Functioning of response options**			
# and % items correctly ordered	7	14	18
	53.8%	56.0%	47.4%
**Item fit**			
Misfitting items	Word recognition	None	Word recognition
**Local dependency**			
Item pairs with *r*≥0.30	2	6	12
**Reliability**			
PSI with extremes	0.88	0.85	0.92

Abbreviations: ADAS‐Cog, AD Assessment Scale‐Cognitive subscale; ADCS‐iADL, AD Cooperative Study‐Instrumental Activities of Daily Living; iADRS, integrated Alzheimer's Disease Rating Scale; PSI, person‐separation index.

#### 3.2.5 RMT analyses of the iADRS components

RMT analyses performed on the ADAS‐Cog_13_ and ADCS‐iADL investigated the measurement properties of the individual component scales. Results showed that the measurement properties of the iADRS, described above, were consistent with those of the ADAS‐Cog_13_ and ADCS‐iADL, providing supportive evidence towards the iADRS total score as an integrated scale. A summary of results is available in Table 4.

Anchored analyses were performed to investigate the mapping of the cognitive items of the iADRS to the ADCS‐iADL and of the daily functioning items to the ADAS‐Cog. Results were comparable to the unanchored analyses, indicating that cognitive and functional item sets performed as expected when incorporated into a single integrated scale.

#### 3.2.6 Crosswalks

A crosswalk, or conversion table, between the iADRS, MMSE, and CDR‐SB was developed. The crosswalk enabled direct score conversions from one test to the other by creating a common metric through test equating.^46^, ^47^ In this way, patients with a known score on one test have an expected corresponding score on the other tests.

A crosswalk was developed using a training set of 24,156 patient/visit records and validated with a testing set of 6038 records, shown to have similar characteristics and scores on the iADRS, MMSE, CDR‐SB, and Global CDR. Validation of the crosswalk was performed by testing the agreement between observed scores and crosswalk‐predicted scores using ICC(A,1) with 95% confidence intervals. Table S4 presents the mean observed score, mean predicted score, mean difference between observed and predicted, and ICCs between the training and validation samples. All comparisons met the threshold of ≥0.70 for between group differences except the iADRS to CDR‐SB crosswalk in the validation set, which had an ICC of 0.69, falling just short of the threshold for adequate reliability (ICC = 0.82 in the training dataset). Overall, the results from the validation set confirm that the crosswalk provides an accurate translation from one score to the others.

In the crosswalk, provided in Table S5, an MMSE stage of “mild impairment” (MMSE score 20–26) equates to an iADRS scores of about 92–129, an MMSE stage of “moderate impairment” (MMSE score 10–19) equates to an iADRS score of about 16–91, and an MMSE stage of “severe impairment” (MMSE score ≤9) equates to an iADRS score of ≤15. A CDR‐SB stage of “mild dementia” (CDR‐SB score 4.5–9.0) corresponds to an iADRS score of about 32–104, a CDR‐SB stage of “moderate dementia” (CDR‐SB score 9.5–15.5) corresponds to an iADRS score of about 3–31, and a CDR‐SB stage of “severe dementia” (CDR‐SB score 16.0–18.0) corresponds to an iADRS score from 0 to 3.

## 4 DISCUSSION

Findings provide robust psychometric evaluation of the iADRS using data from three recent RCTs in patients with biomarker‐confirmed early symptomatic AD at baseline and followed through 18 months of disease progression. Across the three trials, the iADRS total score exhibited good internal consistency reliability (Cronbach's alpha 0.79–0.84). The iADRS demonstrated clear separation between disease stage classifications defined by the CDR‐SB and MMSE, as well as moderate correlations with both the CDR‐SB and MMSE, providing evidence for construct validity of the iADRS as an assessment of global disease severity. The iADRS also demonstrated good ability to detect worsening over time, with large effect sizes observed in participants who showed clinical decline on other standard measures of disease progression (CDR‐SB and MMSE). Taken together, these findings support the iADRS as a reliable and valid measure capable of detecting changes in cognitive and functional ability for early symptomatic AD patients.

RMT psychometric analyses evaluated how well iADRS items assessing cognition and daily function perform as a set, providing estimates of total score reliability, item‐to‐sample targeting, and ordered item difficulty across the continuum of disease severity. Reliability of the full set of iADRS items was excellent (PSI = 0.92) and exceeded the reliability of the individual cognitive and functional component scales (Table 4). The response functioning and fit of items in the ADAS‐Cog_13_ and the ADCS‐iADL were not significantly altered when integrated into a single scale, suggesting that individual measurement properties were preserved. The one item with outlying fit statistics, *word recognition* (Cog7), resonated with previous findings suggesting limitations to the scoring method employed, which places an artificial ceiling on number of recordable errors.^48^

Analyses of targeting (Figure 1) demonstrated that iADRS assessment of cognitive and functional performance was targeted to the abilities of the participant population across the range of early symptomatic AD. All levels of impairment were covered by the iADRS, with a good distribution of cognitive and functional items along the continuum.

Test equating curves for iADRS cognitive component, the ADAS‐Cog_13_ and functional component, the ADCS‐iADL (Figure 3) demonstrated concurrent, integrated measurement of cognition and function. This finding was complemented by the RMT item hierarchy demonstrating overlapping and interleaved coverage of cognitive and functional impairments across the measurement continuum (Table 3). iADRS items measuring cognition and daily function were interwoven across the hierarchy (Figure 2), with impairments in episodic memory (*talking about reading after 1 h or more; delayed word recall)* proceeding broader declines in immediate memory (*word recall*) and iADLs (*shopping*). More widespread deficits in cognition (*orientation*) and iADL functioning (*finding belongings*, *keeping appointments*) emerged next, followed by multi‐domain cognitive impairments in language (*spoken language ability; comprehension)* and declines in functional independence (*ability to be left home alone*).

A strength of the current work is increased stability of the results achieved through pooling of blinded patient‐level data collected over 18 months in three RCTS in early symptomatic AD. Advantages to conducting psychometric analyses using data collected in clinical trials include biomarker confirmation of AD disease pathology for all participants, application of clear inclusion/exclusion characterizing the population of interest, and enhanced quality control of COA data achieved through required rater training, certification, and in‐trial monitoring. To facilitate clinical interpretation of clinical trial results and individual change over time, RCT data was further leveraged to generate crosswalks between iADRS, CDR‐SB, and MMSE using Rasch analysis. The resulting crosswalks (see Table S5) provide estimated score conversions (i.e., score equivalence) between all three scales across the spectrum of symptomatic AD disease severity, allowing translation of findings between assessments used in RCTs and clinical practice. While previous work provided a method for calculating iADRS based on observed MMSE using linear regression,^49^ bi‐directional crosswalks between CDR‐SB, Iadrs, and MMSE represent a unique contribution to the literature.

## 5 LIMITATIONS

Despite the advantages of leveraging data from RCTs for the present analyses, there are some limitations as well. First, findings may not be fully generalizable to real‐world clinical practice due to exclusion of participants with significant comorbidities and reduced representation of non‐white participants and low socioeconomic status in clinical trials. Consequently, application of findings and use of measurement crosswalks in clinical practice should proceed with this caveat in mind. Second, the nature of psychometric analyses performed may be limited by the RCT design or visit schedule. In the present study, assessment of iADRS test–retest reliability was not feasible due to the length of time between visits. In the RMT analyses, low response frequencies for some response options, though expected based on the stage of disease assessed, may have impacted item calibration, reducing precision. Finally, although present findings provide robust characterization of the iADRS in individuals with early symptomatic AD, inclusive of MCI or mild stage AD at Baseline, results may not fully generalize to a wider range of disease severity.

## 6 CONCLUSIONS AND IMPLICATIONS

The iADRS is an integrated assessment of cognition and daily function that provides a single summary measure of global disease severity in early symptomatic AD. The analyses presented here document the reliability, construct validity, and ability to detect change of the iADRS along with other important evidence attesting to the sound measurement properties of the scale. Together, they provide supportive evidence that the iADRS is a well‐defined instrument that is, fit‐for‐purpose to evaluate a treatment benefit in clinical studies of early symptomatic AD. Finally, crosswalk tables providing estimated score equivalence between the iADRS, CDR‐SB, and MMSE offer a valuable tool facilitating interpretation of clinical trial findings in clinical practice and opening new opportunities for research combining data from diverse data sets.

## AUTHOR CONTRIBUTIONS

All authors contributed to the development of and approved the final manuscript. All authors contributed to research design. S.M., K.C., J.M., and S.C. contributed to data analysis and interpretation. The authors thank Giulia Tronchin, PhD, for comments on an earlier version of the manuscript.

## CONFLICT OF INTEREST STATEMENT

The authors declare no conflict of interest. All author disclosures are available in Supporting Information.

## ETHICS STATMENT

Ethics approval and consent to participate: Study documents, including the protocol, demographic and health information form, interview guide, screener, and informed consent and assent forms received ethical approval from institutional review boards at each study site. All participants provided informed consent to participate in the study.

## Supporting information



Supporting Information

Supporting Information

## Data Availability

The datasets generated during and/or analyzed during the current study are available from the corresponding author on reasonable request.
