# Supplementary material for: The iADRS as an integrated measure of cognition and function: Psychometric evidence from recent clinical trials in early symptomatic Alzheimer's disease
Source: Alzheimers Dement. 2025 Sep 14;21(9):e70656. doi: 10.1002/alz.70656 (PMC12433759; doi:10.1002/alz.70656)
Supplement: Supplementary file 1 — Supporting Information [file ALZ-21-e70656-s002.docx]

# Supplemental Materials

Supplementary Table 1**:** Distribution of baseline iADRS total score according to CDR-SB and MMSE severity subgroups in TRAILBLAZER-ALZ, TRAILBLAZER-ALZ 2, and EXPEDITION-3, per study

|  | **TRAILBLAZER-ALZ**  **(N=245)** | | **TRAILBLAZER-ALZ 2**  **(N-1687)** | | **EXPEDITION-3**  **(N=2124)** | |
| --- | --- | --- | --- | --- | --- | --- |
| **CDR-SB** | **N** | **Mean (SD) iADRS Total Score at Baseline** | **N** | **Mean (SD) iADRS Total Score at Baseline** | **N** | **Mean (SD) iADRS Total Score at Baseline** |
| **Clinical staging (CDR-SB score)** |  |  |  |  |  |  |
| Normal (0) | 0 | - | 4 | 125.25 (9.32) | 6 | 120.67 (7.79) |
| MCI or very mild dementia (0.2-4.0) | 172 | 110.91 (9.81) | 1014 | 110.46 (10.34) | 1311 | 109.71 (10.50) |
| Mild dementia (4.5-9.0) | 71 | 95.65 (11.93) | 621 | 94.91 (11.84) | 775 | 95.02 (12.85) |
| Moderate dementia (9.5-15.5) | 2 | 73.50 (9.19) | 31 | 72.93 (13.86) | 26 | 73.88 (12.45) |
| p-value for between-group comparisons* | <0.0001 | | <0.0001 | | <0.0001 | |
| **MMSE** | **N** | **Mean (SD) iADRS Total Score at Baseline** | **N** | **Mean (SD) iADRS Total Score at Baseline** | **N** | **Mean (SD) iADRS Total Score at Baseline** |
| **Clinical staging (MMSE score)** |  |  |  |  |  |  |
| MCI or very mild dementia (27-30) | 45 | 114.36 (10.44) | 263 | 117.50 (9.23) | 183 | 115.63 (9.71) |
| Mild dementia (20-26) | 176 | 105.88 (11.15) | 1009 | 105.55 (11.12) | 1679 | 104.32 (13.12) |
| Moderate dementia (10-19) | 15 | 87.60 (14.47) | 400 | 91.70 (13.14) | 255 | 92.91 (13.19) |
| Severe dementia (0-9) | 0 | - | 3 | 69.67 (22.50) | 0 | - |
| p-value for between-group comparisons* | <0.0001 | | <0.0001 | | <0.0001 | |

CDR-SB=Clinical Dementia Rating sum of boxes; iADRS=integrated Alzheimer’s Disease Rating Scale; MCI=mild cognitive impairment; MMSE=Mini-Mental State Examination; SD=standard deviation

*ANOVA p-value for between-group comparisons

Supplementary Table 2: Location, in logits, of mean and individual response thresholds by iADRS item

| Subtest | Code | Mean Location | Thr 1 | Thr 2 | Thr 3 | Thr 4 | Thr 5 | Thr 6 | Thr 7 | Thr 8 | Thr 9 | Thr 10 | Thr 11 | Thr 12 |
| --- | --- | --- | --- | --- | --- | --- | --- | --- | --- | --- | --- | --- | --- | --- |
| ADAS-Cog | Cog1 | 1.106 | -0.791 | -0.442 | 0.137 | 0.830 | 1.521 | 2.094 | 2.434 | 2.425 | 1.952 | 0.899 |  |  |
| ADAS-Cog | Cog2 | -0.629 | -0.881 | -0.871 | -0.858 | -0.615 | 0.079 |  |  |  |  |  |  |  |
| ADAS-Cog | Cog3 | -0.705 | -1.701 | -0.881 | -0.570 | -0.393 | 0.021 |  |  |  |  |  |  |  |
| ADAS-Cog | Cog4 | -0.493 | -2.114 | -0.626 | -0.713 | -0.581 | 1.567 |  |  |  |  |  |  |  |
| ADAS-Cog | Cog5 | -0.395 | 0.085 | -0.586 | -0.820 | -0.629 | -0.026 |  |  |  |  |  |  |  |
| ADAS-Cog | Cog6 | 0.223 | -2.024 | -0.777 | -0.022 | 0.394 | 0.621 | 0.809 | 1.111 | 1.676 |  |  |  |  |
| ADAS-Cog | Cog7 | 1.184 | 0.425 | 0.422 | 0.459 | 0.537 | 0.658 | 0.821 | 1.029 | 1.282 | 1.580 | 1.924 | 2.316 | 2.756 |
| ADAS-Cog | Cog8 | -1.048 | -2.099 | -1.024 | -0.401 | -0.425 | -1.290 |  |  |  |  |  |  |  |
| ADAS-Cog | Cog9 | -1.183 | -2.442 | -1.344 | -0.757 | -0.596 | -0.776 |  |  |  |  |  |  |  |
| ADAS-Cog | Cog10 | -0.735 | -2.597 | -0.770 | -0.259 | -0.225 | 0.175 |  |  |  |  |  |  |  |
| ADAS-Cog | Cog11 | -0.343 | 0.485 | -0.509 | -0.284 | -0.119 | -1.290 |  |  |  |  |  |  |  |
| ADAS-Cog | Cog12 | 1.932 | 1.337 | 1.323 | 1.442 | 1.649 | 1.899 | 2.149 | 2.353 | 2.468 | 2.449 | 2.252 |  |  |
| ADAS-Cog | Cog13 | 1.232 | 0.026 | 0.373 | 1.305 | 2.164 | 2.293 |  |  |  |  |  |  |  |
| ADCS-iADL | DL6A | -0.386 | 1.428 | -1.484 | -1.101 |  |  |  |  |  |  |  |  |  |
| ADCS-iADL | DL7 | 0.302 | 0.359 | -1.814 | -0.066 | 2.050 | 0.984 |  |  |  |  |  |  |  |
| ADCS-iADL | DL9 | -0.523 | -0.354 | 0.246 | -1.459 |  |  |  |  |  |  |  |  |  |
| ADCS-iADL | DL10 | -0.427 | 1.863 | -1.473 | -1.671 |  |  |  |  |  |  |  |  |  |
| ADCS-iADL | DL11 | -0.092 | -0.663 | 0.053 | 0.335 |  |  |  |  |  |  |  |  |  |
| ADCS-iADL | DL12 | -0.552 | -1.474 | 1.712 | -1.894 |  |  |  |  |  |  |  |  |  |
| ADCS-iADL | DL13 | 0.104 | 0.438 | -0.053 | 1.415 | -1.386 |  |  |  |  |  |  |  |  |
| ADCS-iADL | DL14 | -0.595 | 1.422 | -1.823 | -1.383 |  |  |  |  |  |  |  |  |  |
| ADCS-iADL | DL15 | -0.471 | -0.380 | -2.404 | 0.807 | 0.094 |  |  |  |  |  |  |  |  |
| ADCS-iADL | DL16 | 0.232 | 0.777 | -0.562 | 0.447 | 0.266 |  |  |  |  |  |  |  |  |
| ADCS-iADL | DL17 | -0.144 | -1.356 | -0.024 | 0.949 |  |  |  |  |  |  |  |  |  |
| ADCS-iADL | DL8A | -0.592 | -0.592 |  |  |  |  |  |  |  |  |  |  |  |
| ADCS-iADL | DL8B | 0.088 | 0.088 |  |  |  |  |  |  |  |  |  |  |  |
| ADCS-iADL | DL8C | 1.611 | 1.611 |  |  |  |  |  |  |  |  |  |  |  |
| ADCS-iADL | DL18A | 0.022 | 0.022 |  |  |  |  |  |  |  |  |  |  |  |
| ADCS-iADL | DL18B | -1.047 | -1.047 |  |  |  |  |  |  |  |  |  |  |  |
| ADCS-iADL | DL18C | -1.288 | -1.288 |  |  |  |  |  |  |  |  |  |  |  |
| ADCS-iADL | DL19A | -0.157 | -0.157 |  |  |  |  |  |  |  |  |  |  |  |
| ADCS-iADL | DL19B | 0.075 | 0.075 |  |  |  |  |  |  |  |  |  |  |  |
| ADCS-iADL | DL19C | -0.102 | -0.102 |  |  |  |  |  |  |  |  |  |  |  |
| ADCS-iADL | DL20A | 1.017 | 1.017 |  |  |  |  |  |  |  |  |  |  |  |
| ADCS-iADL | DL20B | 2.095 | 2.095 |  |  |  |  |  |  |  |  |  |  |  |
| ADCS-iADL | DL21 | 0.753 | -0.171 | -0.317 | 2.748 |  |  |  |  |  |  |  |  |  |
| ADCS-iADL | DL22 | -0.003 | 2.365 | -1.203 | -1.170 |  |  |  |  |  |  |  |  |  |
| ADCS-iADL | DL23 | -0.069 | 1.865 | -1.081 | -0.614 | -0.445 |  |  |  |  |  |  |  |  |

Thr = threshold boundary

Supplementary Table 3: iADRS residual correlations in TRAILBLAZER-ALZ, TRAILBLAZER-ALZ 2, and EXPEDITION-3

| **Item** | **Cog1** | **Cog2** | **Cog3** | **Cog4** | **Cog5** | **Cog6** | **Cog7** | **Cog8** | **Cog9** | **Cog10** | **Cog11** | **Cog12** | **Cog13** | **DL6A** | **DL7** | **DL9** | **DL10** | **DL11** | **DL12** | **DL13** | **DL14** | **DL15** | **DL16** | **DL17** | **DL8A** | **DL8B** | **DL8C** | **DL18A** | **DL18B** | **DL18C** | **DL19A** | **DL19B** | **DL19C** | **DL20A** | **DL20B** | **DL21** | **DL22** | **DL23** |
| --- | --- | --- | --- | --- | --- | --- | --- | --- | --- | --- | --- | --- | --- | --- | --- | --- | --- | --- | --- | --- | --- | --- | --- | --- | --- | --- | --- | --- | --- | --- | --- | --- | --- | --- | --- | --- | --- | --- |
| **Cog1** | 1.00 |  |  |  |  |  |  |  |  |  |  |  |  |  |  |  |  |  |  |  |  |  |  |  |  |  |  |  |  |  |  |  |  |  |  |  |  |  |
| **Cog2** | 0.18 | 1.00 |  |  |  |  |  |  |  |  |  |  |  |  |  |  |  |  |  |  |  |  |  |  |  |  |  |  |  |  |  |  |  |  |  |  |  |  |
| **Cog3** | 0.12 | 0.17 | 1.00 |  |  |  |  |  |  |  |  |  |  |  |  |  |  |  |  |  |  |  |  |  |  |  |  |  |  |  |  |  |  |  |  |  |  |  |
| **Cog4** | 0.00 | 0.04 | 0.13 | 1.00 |  |  |  |  |  |  |  |  |  |  |  |  |  |  |  |  |  |  |  |  |  |  |  |  |  |  |  |  |  |  |  |  |  |  |
| **Cog5** | 0.03 | 0.10 | 0.15 | 0.19 | 1.00 |  |  |  |  |  |  |  |  |  |  |  |  |  |  |  |  |  |  |  |  |  |  |  |  |  |  |  |  |  |  |  |  |  |
| **Cog6** | 0.02 | -0.02 | -0.07 | -0.06 | -0.01 | 1.00 |  |  |  |  |  |  |  |  |  |  |  |  |  |  |  |  |  |  |  |  |  |  |  |  |  |  |  |  |  |  |  |  |
| **Cog7** | 0.05 | -0.07 | -0.09 | -0.13 | -0.12 | 0.01 | 1.00 |  |  |  |  |  |  |  |  |  |  |  |  |  |  |  |  |  |  |  |  |  |  |  |  |  |  |  |  |  |  |  |
| **Cog8** | 0.07 | 0.13 | 0.10 | 0.03 | 0.05 | -0.05 | -0.08 | 1.00 |  |  |  |  |  |  |  |  |  |  |  |  |  |  |  |  |  |  |  |  |  |  |  |  |  |  |  |  |  |  |
| **Cog9** | 0.08 | 0.12 | 0.17 | 0.05 | 0.08 | -0.02 | -0.07 | 0.42 | 1.00 |  |  |  |  |  |  |  |  |  |  |  |  |  |  |  |  |  |  |  |  |  |  |  |  |  |  |  |  |  |
| **Cog10** | 0.13 | 0.20 | 0.15 | 0.06 | 0.08 | -0.07 | -0.08 | 0.48 | 0.36 | 1.00 |  |  |  |  |  |  |  |  |  |  |  |  |  |  |  |  |  |  |  |  |  |  |  |  |  |  |  |  |
| **Cog11** | 0.09 | 0.04 | 0.06 | -0.01 | 0.02 | 0.00 | 0.12 | 0.06 | 0.16 | 0.11 | 1.00 |  |  |  |  |  |  |  |  |  |  |  |  |  |  |  |  |  |  |  |  |  |  |  |  |  |  |  |
| **Cog12** | 0.28 | -0.03 | -0.11 | -0.13 | -0.11 | 0.16 | 0.16 | -0.11 | -0.09 | -0.09 | -0.02 | 1.00 |  |  |  |  |  |  |  |  |  |  |  |  |  |  |  |  |  |  |  |  |  |  |  |  |  |  |
| **Cog13** | 0.11 | 0.08 | 0.16 | 0.23 | 0.14 | -0.08 | -0.14 | 0.04 | 0.06 | 0.10 | 0.03 | -0.12 | 1.00 |  |  |  |  |  |  |  |  |  |  |  |  |  |  |  |  |  |  |  |  |  |  |  |  |  |
| **DL6A** | -0.11 | -0.02 | -0.06 | -0.05 | -0.03 | -0.08 | -0.14 | -0.07 | -0.08 | -0.10 | -0.09 | -0.10 | -0.06 | 1.00 |  |  |  |  |  |  |  |  |  |  |  |  |  |  |  |  |  |  |  |  |  |  |  |  |
| **DL7** | -0.14 | -0.06 | -0.07 | -0.06 | -0.04 | -0.08 | -0.21 | -0.05 | -0.08 | -0.08 | -0.11 | -0.14 | -0.04 | 0.05 | 1.00 |  |  |  |  |  |  |  |  |  |  |  |  |  |  |  |  |  |  |  |  |  |  |  |
| **DL9** | -0.10 | -0.04 | -0.04 | -0.07 | -0.06 | -0.12 | -0.10 | -0.04 | -0.06 | -0.04 | -0.04 | -0.11 | -0.09 | 0.05 | 0.03 | 1.00 |  |  |  |  |  |  |  |  |  |  |  |  |  |  |  |  |  |  |  |  |  |  |
| **DL10** | -0.13 | -0.06 | -0.03 | 0.00 | -0.04 | -0.13 | -0.12 | -0.05 | -0.07 | -0.07 | -0.07 | -0.14 | -0.03 | 0.06 | 0.01 | 0.02 | 1.00 |  |  |  |  |  |  |  |  |  |  |  |  |  |  |  |  |  |  |  |  |  |
| **DL11** | -0.16 | -0.11 | -0.09 | -0.04 | -0.06 | -0.11 | -0.11 | -0.08 | -0.09 | -0.09 | -0.10 | -0.11 | -0.07 | 0.10 | 0.02 | 0.09 | 0.11 | 1.00 |  |  |  |  |  |  |  |  |  |  |  |  |  |  |  |  |  |  |  |  |
| **DL12** | -0.14 | -0.08 | -0.05 | -0.01 | -0.03 | -0.10 | -0.10 | -0.05 | -0.06 | -0.07 | -0.08 | -0.13 | -0.04 | 0.01 | 0.03 | -0.03 | 0.08 | 0.00 | 1.00 |  |  |  |  |  |  |  |  |  |  |  |  |  |  |  |  |  |  |  |
| **DL13** | -0.18 | -0.09 | -0.07 | -0.08 | -0.06 | -0.14 | -0.16 | -0.09 | -0.12 | -0.12 | -0.12 | -0.16 | -0.08 | 0.19 | 0.03 | 0.00 | 0.13 | 0.06 | 0.15 | 1.00 |  |  |  |  |  |  |  |  |  |  |  |  |  |  |  |  |  |  |
| **DL14** | -0.14 | -0.04 | -0.06 | -0.05 | -0.04 | -0.09 | -0.11 | -0.06 | -0.06 | -0.07 | -0.07 | -0.11 | -0.05 | 0.06 | 0.01 | 0.03 | 0.13 | 0.13 | 0.02 | 0.08 | 1.00 |  |  |  |  |  |  |  |  |  |  |  |  |  |  |  |  |  |
| **DL15** | -0.21 | -0.09 | -0.10 | -0.01 | -0.07 | -0.04 | -0.19 | -0.09 | -0.10 | -0.12 | -0.08 | -0.14 | -0.06 | -0.02 | 0.07 | -0.03 | 0.02 | -0.02 | 0.05 | 0.02 | 0.02 | 1.00 |  |  |  |  |  |  |  |  |  |  |  |  |  |  |  |  |
| **DL16** | -0.24 | -0.10 | -0.12 | -0.04 | -0.07 | -0.11 | -0.21 | -0.10 | -0.11 | -0.12 | -0.11 | -0.17 | -0.11 | 0.08 | 0.07 | 0.03 | 0.07 | 0.08 | 0.04 | 0.08 | 0.07 | 0.25 | 1.00 |  |  |  |  |  |  |  |  |  |  |  |  |  |  |  |
| **DL17** | -0.21 | -0.10 | -0.13 | -0.10 | -0.08 | 0.03 | -0.20 | -0.11 | -0.11 | -0.18 | -0.11 | -0.06 | -0.14 | 0.07 | 0.07 | 0.05 | 0.02 | 0.13 | -0.02 | 0.04 | 0.07 | 0.10 | 0.14 | 1.00 |  |  |  |  |  |  |  |  |  |  |  |  |  |  |
| **DL8A** | -0.09 | -0.08 | -0.08 | -0.11 | -0.08 | -0.05 | -0.12 | -0.05 | -0.05 | -0.08 | -0.07 | -0.03 | -0.09 | 0.03 | 0.06 | 0.05 | 0.01 | 0.03 | 0.00 | 0.03 | 0.01 | 0.04 | 0.04 | 0.09 | 1.00 |  |  |  |  |  |  |  |  |  |  |  |  |  |
| **DL8B** | -0.12 | -0.07 | -0.06 | -0.07 | -0.09 | -0.12 | -0.11 | -0.06 | -0.06 | -0.06 | -0.07 | -0.09 | -0.09 | -0.01 | 0.03 | 0.13 | 0.01 | 0.00 | 0.01 | -0.01 | 0.02 | -0.01 | 0.02 | 0.04 | 0.20 | 1.00 |  |  |  |  |  |  |  |  |  |  |  |  |
| **DL8C** | -0.15 | -0.11 | -0.14 | -0.15 | -0.14 | -0.02 | -0.10 | -0.09 | -0.09 | -0.12 | -0.07 | -0.02 | -0.19 | 0.00 | 0.05 | 0.09 | -0.02 | 0.03 | -0.01 | -0.01 | 0.01 | 0.03 | 0.05 | 0.13 | 0.19 | 0.28 | 1.00 |  |  |  |  |  |  |  |  |  |  |  |
| **DL18A** | -0.17 | -0.10 | -0.10 | -0.05 | -0.08 | -0.03 | -0.19 | -0.06 | -0.06 | -0.10 | -0.07 | -0.10 | -0.08 | 0.00 | 0.09 | -0.02 | 0.00 | -0.01 | 0.05 | 0.02 | -0.02 | 0.46 | 0.18 | 0.07 | 0.08 | 0.00 | 0.06 | 1.00 |  |  |  |  |  |  |  |  |  |  |
| **DL18B** | -0.12 | -0.08 | -0.07 | -0.08 | -0.07 | -0.05 | -0.14 | -0.06 | -0.05 | -0.09 | -0.07 | -0.08 | -0.07 | 0.02 | 0.06 | -0.01 | 0.02 | 0.02 | 0.06 | 0.04 | 0.01 | 0.18 | 0.04 | 0.05 | 0.07 | 0.01 | 0.04 | 0.34 | 1.00 |  |  |  |  |  |  |  |  |  |
| **DL18C** | -0.11 | -0.07 | -0.06 | -0.06 | -0.06 | -0.05 | -0.11 | -0.06 | -0.04 | -0.08 | -0.06 | -0.08 | -0.06 | 0.01 | 0.04 | 0.00 | 0.03 | 0.01 | 0.05 | 0.04 | 0.01 | 0.15 | 0.02 | 0.02 | 0.05 | 0.01 | 0.02 | 0.32 | 0.71 | 1.00 |  |  |  |  |  |  |  |  |
| **DL19A** | -0.14 | -0.07 | -0.08 | -0.14 | -0.12 | -0.07 | -0.15 | -0.07 | -0.08 | -0.10 | -0.08 | -0.06 | -0.14 | 0.00 | 0.03 | 0.12 | -0.02 | 0.00 | -0.02 | 0.00 | 0.03 | 0.03 | 0.02 | 0.11 | 0.17 | 0.28 | 0.29 | 0.04 | 0.04 | 0.02 | 1.00 |  |  |  |  |  |  |  |
| **DL19B** | -0.17 | -0.10 | -0.12 | -0.16 | -0.12 | -0.06 | -0.16 | -0.09 | -0.10 | -0.13 | -0.09 | -0.07 | -0.16 | 0.01 | 0.06 | 0.14 | -0.01 | 0.02 | -0.02 | 0.01 | 0.04 | 0.07 | 0.08 | 0.15 | 0.13 | 0.20 | 0.25 | 0.11 | 0.07 | 0.06 | 0.45 | 1.00 |  |  |  |  |  |  |
| **DL19C** | -0.16 | -0.09 | -0.11 | -0.15 | -0.13 | -0.06 | -0.17 | -0.09 | -0.09 | -0.12 | -0.09 | -0.07 | -0.16 | 0.02 | 0.05 | 0.15 | -0.01 | 0.03 | -0.02 | 0.01 | 0.03 | 0.05 | 0.06 | 0.14 | 0.14 | 0.22 | 0.26 | 0.08 | 0.07 | 0.06 | 0.50 | 0.64 | 1.00 |  |  |  |  |  |
| **DL20A** | -0.14 | -0.06 | -0.05 | -0.05 | -0.05 | -0.08 | -0.14 | -0.05 | -0.06 | -0.07 | -0.07 | -0.09 | -0.06 | -0.04 | 0.04 | 0.10 | -0.02 | -0.02 | -0.02 | -0.04 | -0.01 | -0.01 | -0.01 | 0.06 | 0.08 | 0.24 | 0.20 | 0.02 | 0.00 | -0.01 | 0.25 | 0.18 | 0.19 | 1.00 |  |  |  |  |
| **DL20B** | -0.17 | -0.09 | -0.10 | -0.12 | -0.09 | -0.02 | -0.14 | -0.07 | -0.08 | -0.11 | -0.09 | -0.05 | -0.14 | -0.01 | 0.06 | 0.08 | -0.04 | 0.00 | -0.02 | -0.02 | 0.01 | 0.03 | 0.04 | 0.13 | 0.09 | 0.17 | 0.36 | 0.07 | 0.03 | 0.01 | 0.23 | 0.21 | 0.22 | 0.48 | 1.00 |  |  |  |
| **DL21** | -0.11 | -0.02 | -0.01 | 0.04 | 0.03 | -0.09 | -0.22 | -0.03 | -0.05 | -0.04 | -0.06 | -0.17 | 0.01 | 0.03 | 0.11 | 0.03 | 0.01 | 0.01 | 0.00 | 0.01 | 0.01 | 0.05 | 0.07 | 0.10 | 0.01 | 0.03 | 0.01 | 0.07 | 0.06 | 0.05 | 0.02 | 0.06 | 0.05 | 0.07 | 0.07 | 1.00 |  |  |
| **DL22** | -0.13 | -0.07 | -0.04 | 0.02 | -0.01 | -0.09 | -0.09 | -0.06 | -0.06 | -0.07 | -0.06 | -0.11 | -0.01 | -0.01 | -0.03 | 0.04 | 0.02 | 0.03 | -0.02 | -0.01 | 0.01 | 0.01 | 0.00 | 0.01 | 0.00 | 0.03 | 0.01 | 0.00 | -0.01 | 0.00 | 0.03 | 0.03 | 0.02 | 0.09 | 0.05 | 0.04 | 1.00 |  |
| **DL23** | -0.21 | -0.08 | -0.07 | -0.03 | -0.03 | -0.14 | -0.18 | -0.09 | -0.10 | -0.12 | -0.10 | -0.19 | -0.06 | 0.08 | 0.05 | 0.00 | 0.12 | 0.07 | 0.17 | 0.24 | 0.09 | 0.07 | 0.10 | 0.06 | 0.01 | 0.00 | -0.02 | 0.06 | 0.07 | 0.06 | 0.00 | 0.02 | 0.00 | 0.00 | -0.01 | 0.03 | 0.02 | 1.00 |

Note: highlighted items are correlations ≥0.30

Supplementary Table 4: ICCs between observed scores and scores predicted by crosswalk, using pooled and stacked data from TRAILBLAZER-ALZ, TRAILBLAZER-ALZ 2, and EXPEDITION-3

|  | | **n^1^** | Mean (SD) **Observed Score** | Mean (SD) **Predicted Score** | Mean (SD) **Difference** | **ICC**^2^ **(95% CI)** |
| --- | --- | --- | --- | --- | --- | --- |
|  |  |  |  |  |  |  |
| iADRS score, as predicted by the CDR‑SB to iADRS crosswalk | Training Set | n=12,441 | 100.4 (17.8) | 99.8 (35.6) | -0.7 (24.2) | **0.85** (0.83-0.86) |
|  | Validation Set | n=3,062 | 100.1 (18.2) | 99.5 (36.3) | -0.7 (24.2) | **0.74** (0.72-0.76) |
| iADRS score, as predicted by the MMSE to iADRS crosswalk | Training Set | n=16,645 | 99.1 (18.9) | 100.0 (29.2) | 0.9 (19.5) | **0.88** (0.85-0.90) |
|  | Validation Set | n=4,158 | 98.9 (19.1) | 99.7 (29.5) | 0.7 (19.6) | **0.75** (0.73-0.77) |
| CDR-SB score, as predicted by the iADRS to CDR‑SB crosswalk | Training Set | n=12,441 | 4.65 ( 2.7) | 4.64 ( 1.1) | -0.05 ( 1.9) | **0.82** (0.79-0.83) |
|  | Validation Set | n=3,062 | 4.68 ( 2.7) | 4.65 ( 1.1) | -0.06 ( 1.9) | **0.69** (0.66-0.71) |
| CDR-SB score, as predicted by the MMSE to CDR-SB crosswalk | Training Set | n=12,454 | 4.64 ( 2.7) | 4.53 ( 2.1) | -0.12 ( 2.1) | **0.83** (0.80-0.85) |
|  | Validation Set | n=3,069 | 4.67 ( 2.7) | 4.57 ( 2.1) | -0.10 ( 2.1) | **0.73** (0.70-0.75) |
| MMSE score, as predicted by the iADRS to MMSE crosswalk | Training Set | n=16,645 | 21.2 ( 4.7) | 20.8 ( 3.1) | -0.4 ( 3.2) | **0.89** (0.86-0.90) |
|  | Validation Set | n=4,158 | 21.2 ( 4.8) | 20.8 ( 3.1) | -0.4 ( 3.2) | **0.76** (0.74-0.78) |
| MMSE score, as predicted by the CDR-SB to MMSE crosswalk | Training Set | n=12,454 | 21.5 ( 4.6) | 21.4 ( 5.7) | -0.1 ( 4.5) | **0.85** (0.82-0.86) |
|  | Validation Set | n=3,069 | 21.4 ( 4.6) | 21.3 ( 5.8) | -0.1 ( 4.5) | **0.73** (0.71-0.75) |

^1^ n = number of observations (one observation per participant, per visit)

^2^ ICC(A,1) = two-way random effects with absolute agreement and a single measurement approach, according to *McGraw and Wong 1996 Convention [39]*

Supplementary Table 5: Crosswalk between iADRS, MMSE and CDR-SB using pooled and stacked data from TRAILBLAZER-ALZ, TRAILBLAZER-ALZ 2, and EXPEDITION-3

| **iADRS** | **MMSE** | **CDR-SB** |  | **iADRS** | **MMSE** | **CDR-SB** |  | **iADRS** | **MMSE** | **CDR-SB** |
| --- | --- | --- | --- | --- | --- | --- | --- | --- | --- | --- |
|  | 0 |  |  | 21 |  |  |  | 48 | 14 | 8 |
|  |  | 18 |  | 22 |  |  |  | 49 |  |  |
| 0 |  |  |  | 23 |  |  |  | 50 |  |  |
|  | 1 |  |  | 24 |  |  |  | 51 |  |  |
|  |  | 17 |  | 25 |  |  |  | 52 |  |  |
| 1 | 2 |  |  | 26 |  |  |  | 53 |  |  |
| 2 | 3 |  |  |  | 11 | 10 |  | 54 |  |  |
|  |  | 16 |  | 27 |  |  |  | 55 |  |  |
| 3 | 4 |  |  | 28 |  |  |  | 56 |  |  |
| 4 |  | 15 |  | 29 |  |  |  | 57 |  |  |
|  | 5 |  |  | 30 |  |  |  | 58 |  |  |
| 5 |  |  |  | 31 |  |  |  | 59 |  |  |
| 6 | 6 | 14 |  | 32 |  |  |  | 60 |  |  |
| 7 |  |  |  |  | 12 |  |  | 61 | 15 | 7 |
| 8 | 7 |  |  | 33 |  |  |  | 62 |  |  |
| 9 |  | 13 |  | 34 |  |  |  | 63 |  |  |
| 10 |  |  |  | 35 |  |  |  | 64 |  |  |
| 11 |  |  |  | 36 |  | 9 |  | 65 |  |  |
|  | 8 |  |  | 37 |  |  |  | 66 |  |  |
| 12 |  |  |  | 38 |  |  |  | 67 |  |  |
|  |  | 12 |  | 39 |  |  |  | 68 |  |  |
| 13 |  |  |  |  | 13 |  |  | 69 |  |  |
| 14 |  |  |  | 40 |  |  |  |  | 16 |  |
| 15 |  |  |  | 41 |  |  |  | 70 |  |  |
|  | 9 |  |  | 42 |  |  |  | 71 |  |  |
| 16 |  |  |  | 43 |  |  |  | 72 |  |  |
| 17 |  |  |  | 44 |  |  |  | 73 |  |  |
| 18 |  |  |  | 45 |  |  |  |  |  | 6 |
| 19 | 10 | 11 |  | 46 |  |  |  | 74 |  |  |
| 20 |  |  |  | 47 |  |  |  | 75 |  |  |

Continued on next page…

Supplementary Table 5: Crosswalk between iADRS, MMSE and CDR-SB using pooled and stacked data from TRAILBLAZER-ALZ, TRAILBLAZER-ALZ 2, and EXPEDITION-3 (continued)

| **iADRS** | **MMSE** | **CDR-SB** |  | **iADRS** | **MMSE** | **CDR-SB** |  | **iADRS** | **MMSE** | **CDR-SB** |
| --- | --- | --- | --- | --- | --- | --- | --- | --- | --- | --- |
| 76 |  |  |  | 103 |  |  |  | 129 |  |  |
| 77 |  |  |  | 104 |  |  |  |  | 26 |  |
|  | 17 |  |  |  |  | 4.5 |  | 130 |  |  |
| 78 |  |  |  | 105 |  |  |  | 131 |  |  |
| 79 |  |  |  | 106 |  |  |  | 132 |  |  |
| 80 |  |  |  |  | 22 |  |  |  |  | 2.5 |
| 81 |  |  |  | 107 |  |  |  | 133 |  |  |
| 82 |  |  |  | 108 |  |  |  |  | 27 |  |
| 83 |  |  |  | 109 |  |  |  | 134 |  |  |
| 84 |  |  |  | 110 |  |  |  | 135 |  |  |
|  | 18 | 5.5 |  |  |  | 4 |  | 136 |  |  |
| 85 |  |  |  | 111 |  |  |  | 137 |  |  |
| 86 |  |  |  | 112 |  |  |  |  | 28 | 2 |
| 87 |  |  |  |  | 23 |  |  | 138 |  |  |
| 88 |  |  |  | 113 |  |  |  | 139 |  |  |
| 89 |  |  |  | 114 |  |  |  | 140 |  |  |
| 90 |  |  |  | 115 |  |  |  |  |  | 1.5 |
| 91 | 19 |  |  | 117 | 24 |  |  | 141 | 29 |  |
| 92 |  |  |  | 118 |  |  |  | 142 |  |  |
| 93 |  |  |  | 119 |  |  |  |  |  | 1 |
| 94 |  | 5 |  |  |  | 3.5 |  |  |  |  |
| 95 |  |  |  | 120 |  |  |  | 143 | 30 |  |
| 96 |  |  |  | 121 |  |  |  |  |  | 0.5 |
|  | 20 |  |  | 122 |  |  |  | 144 |  |  |
| 97 |  |  |  | 123 |  |  |  |  |  | 0 |
| 98 |  |  |  | 124 | 25 |  |  |  |  |  |
| 99 |  |  |  | 125 |  |  |  |  |  |  |
| 100 |  |  |  | 126 |  | 3 |  |  |  |  |
| 101 |  |  |  | 127 |  |  |  |  |  |  |
| 102 | 21 |  |  | 128 |  |  |  |  |  |  |
